# The effect of early postnatal auditory stimulation on outcomes in preterm infants

**DOI:** 10.1038/s41390-024-03329-7

**Published:** 2024-06-22

**Authors:** Juliann M. Di Fiore, Gloria Liu, Kenneth A. Loparo, Cynthia F. Bearer

**Affiliations:** 1 Dept of Pediatrics, Case Western Reserve University, Cleveland, OH 44016 USA; 2 Division of Neonatology, Rainbow Babies and Children’s Hospital, Cleveland, OH 44016 USA; 3 ISSACS: Institute for Smart, Secure and Connected Systems, Case Western Reserve University, Cleveland, OH USA

## Abstract

**Abstract:**

Preterm infants are deprived of *in utero* sensory stimulation during the third trimester, an important period of central nervous system development. As a result, maturational trajectories are often reduced in infants born preterm. One such system affected is the brain including the auditory and respiratory control pathways. During normal pregnancy the intrauterine environment attenuates external auditory stimuli while exposing the fetus to filtered maternal voice, intra-abdominal sounds, and external stimuli. In contrast, during the third trimester of development, preterm infants are exposed to a vastly different soundscape including non-attenuated auditory sounds and a lack of womb related stimuli, both of which may affect postnatal brain maturation. Therefore, fostering a nurturing postnatal auditory environment during hospitalization may have a significant impact on related outcomes of preterm infants. Studies using a range of postnatal auditory stimulations have suggested that exposure to sounds or lack thereof can have a significant impact on outcomes. However, studies are inconsistent with sound levels, duration of exposure to auditory stimuli, and the gestational age at which infants are exposed.

**Impact:**

- Auditory stimulation can provide a low cost and low risk intervention to stabilize respiration, improve neuronal maturation and reduce long-term sequelae in preterm infants.
- The potential benefits of auditory stimulation are dependent on the type of sound, the duration of exposure and age at time of exposure.
- Future studies should focus on the optimal type and duration of sound exposure and postnatal developmental window to improve outcomes.

## Introduction

Sound is an important part of everyone’s environment, including fetuses. Sound either enters our external auditory canal to impinge on the tympanic membrane causing auditory stimulation or can vibrate the bones of the skull that cause auditory stimulation of the cochlea. The 7 main characteristics of sound waves are amplitude, wavelength, period, frequency, speed, and timbre, all of which can be affected by the surrounding environment. Thus, the sound environment causing auditory stimulation in the womb is unique.

Preterm infants are deprived of normal *in utero* auditory stimulation during the third trimester, an important period of brain development. As a result, brain volumes and growth trajectories are often reduced in preterm infants compared to healthy fetuses.^1^ During normal pregnancy the intrauterine environment attenuates external auditory stimuli.^2,3^ while exposing the fetus to filtered maternal voice, heart and respiratory sounds, intra-abdominal sounds and sounds from external sources.^4^ In contrast, during a similar gestational period, infants born prematurely are exposed to a vastly different external soundscape including non-attenuated auditory sounds and a lack of womb related stimuli, both of which may have an adverse affect on postnatal brain maturation. Therefore, fostering a postnatal auditory environment simulating *in utero* stimulation in the neonatal intensive care unit (NICU) setting may have a significant positive impact on prematurely related outcomes.

Auditory exposures in the NICU may promote vital functions and processing of stimuli. Simple stimulations such as maternal voice can improve cardiorespiratory stability,^5^ neurodevelopment.^6,7^ and auditory cortex maturation,^8^ as well as reduce pain severity scores.^9^ during critical periods of development. In contrast, auditory overstimulation and/or deprivation can be harmful.^10,11^ Therefore, identifying the appropriate sensory NICU environment is a crucial component of clinical care and resulting postnatal development. This review explores the range of postnatal auditory stimuli being investigated in the field of neonatology and their effects on preterm infant outcomes.

## Womb versus NICU environment

*In utero*, the fetus experiences a dynamic range of auditory stimuli from maternal voice, heart, respiratory and bowel sounds to environmental propagations. Sound levels vary with near term intrauterine recordings and can include intermittent sound bursts of up to 60 to 90 dB.^12,13^ Assimilating the *in utero* environment for preterm infants could play an important role in optimizing postnatal development, but the NICU perpetuates a vastly different sound environment. Unlike the womb, and without the “protective” attenuation from the maternal placenta and surrounding tissue, the preterm infant has direct exposure to extreme variations in sound ranging from excessive noise/overstimulation in open bay NICUs due to ventilators, pagers, bedside alarms, vocalizations, and other sources, to extreme quiet/lack of stimulation in NICUs with single patient rooms.^14^ Current guidelines have focused on preventing sound exposure over 45dB.^15–17^ which is equivalent to a quiet library setting. However, given the high sound levels that may be experienced *in utero*, these guidelines lack recommendations on the appropriate temporal patterns, frequency spectra and sound levels in the NICU to compensate for early preterm birth.

To reduce noise, many NICUs have gone from open bay to single patient rooms showing a significant reduction in noise.^18,19^ and increased time with periods of silence.^19^ Although multiple studies have shown improved outcomes in single patient rooms such as shorter time to establish full enteric feeds.^18^ and increased parental presence and wellbeing,^18,20^ a non-randomized study of 136 preterm infants showed greater impairment in language outcomes and brain development in private rooms, especially in infants with moderate-severe cerebral injury suggesting the potential for a detrimental increase in sensory deprivation.^11^ Thus, single patient rooms may decrease harmful ambient noise, but infants with no or limited parental visits and interaction may need further intervention.

## Postnatal auditory stimuli

**Fig. 1:**
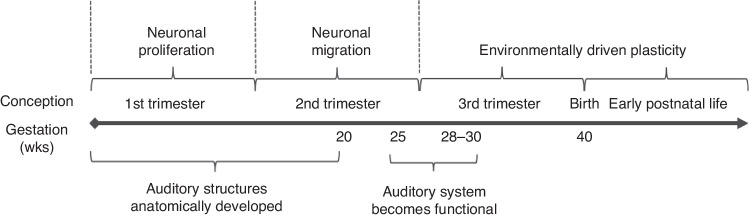
Development of the auditory system.

The fetus can respond to sounds after approximately 26–28 weeks of gestation.^21^ with further maturation of the auditory cortical networks occurring during the last trimester (Fig. 1). Therefore, the early postnatal period following preterm birth presents an important developmental window to enhance auditory induced neuronal maturation. In fact, preliminary data in a small study of 23 infants found lower P1 latencies (the time between initial synapses recorded in a brainstem auditory evoked response (BAER) test) in preterm versus term infants at 3 months corrected age suggesting that earlier prolonged postnatal exposure to auditory stimulation after preterm birth may accelerate development of auditory cortical pathways.^22^ Which, when and how external auditory stimuli enhance the development of the brain and cortical circuitry has yet to be elucidated.

## Types of interventions

### Reducing ambient noise

While some studies have focused on stimulatory therapy, others have assessed the effects of reducing potentially harmful environmental sounds by applying muffs/ear plugs or exposing infants to “white noise.” White noise has no discernable temporal pattern (regularity) and includes a broad range of frequencies at uniform intensity and is therefore dissimilar to what a fetus would experience *in utero*. Reducing sound exposure for short or prolonged periods of time, or attenuating sounds in general, have yielded mixed findings (Table 1). For example, MiniMuffs placed over the infant’s ears 5 min before and during painful procedures improved oxygenation and comfort levels while decreasing pain severity, stress and crying time.^23^ Longer exposures in very low birth weight newborns randomized to wearing earplugs from 1 week of age to 35 weeks corrected age versus normal clinical care showed that earplugs facilitated weight gain at 34 weeks corrected age.^24^ with newborns wearing earplugs weighing an average of 111 g more. In addition, a secondary analysis in a subcohort of the infants <1000 g at birth revealed significantly higher mental developmental scores at 18–22 months of age in the earplug group.^24^

**Table 1:** Auditory stimulation and outcomes in preterm infants.

Intervention	Duration	Sound levels	Population	Results	Reference
**Noise Reduction**					
Silicone earplugs	Time of randomization to 35wks CGA or discharge	Reduced sound levels by 17.7 dB	BW < 1500 g <1wk of age *n* = 24	Increased weight at 34wks CGA; in infants <1000 g, improved MDI scores and head circumference at 18 to 22 months	Abou Turk et al.^24^
MiniMuffs during painful procedure	5 min before and during procedure	Reduced sound levels by ≥7 dB	31–36wks GA *n* = 32	Decreased pain severity, stress, heart rate and crying time; increased comfort levels and oxygen saturation	Kahraman et al.^23^
White noise (light rain)	20 min 3x per day 4 consecutive days	50–55 dB	<37wks GA ≤1800g in incubators *n* = 69	No effect on weight gain, sleep-wake patterns, salivary cortisol levels, oxygen saturation or heart rate	Liao et al.^26^
Noise reduction program (neonates placed in incubators, phone and door ring noises diminished and flashlights used in place of alarms and vibrations)	4 week program	NA^a^	26–36wks GA BW 1000–2500 g *n* = 85	No change in salivary cortisol levels	Gholami et al.^25^
**Maternal/Adult Voice**					
Recorded adult speech	16 h recordings at 32 and 36wks CGA	NA	≤32wks GA ≤ 1250 g *n* = 36	Improved cognitive and language scores at 7 and 18 months CGA	Caskey et al.^6^
Recorded maternal voice	5 min before and during procedure	50 dB	31–36wks GA *n* = 32	Decreased pain severity, stress and crying time, and increased comfort levels and oxygen saturation	Kahraman et al.^23^
Maternal reading through bone conductor device on the wrist	21 days 3 sessions daily 30-32wks CGA	48 dB	<1500 g *n* = 71	Reduced heart rate, promoted stable skin color; improved neurofunctional assessment at 3 months CGA with no difference at 6 months CGA	Picciolini et al.^28^
Maternal Singing					
Recorded maternal-sung lullaby	15 min sessions 3 consecutive days postnatal age of ≥3 days	65–70 dB	29–34wks GA ≥ 3 days postnatal age ≤2800 g weight *n* = 41	Improved oxygen saturation	Jabraeili et al.^31^
Maternal singing during kangaroo care	1.5–2 h a day 15-63 days until 40 wks CGA	NA	26–33wks GA *n* = 45	Increased infant mismatch responsess to phonetic and emotional speech sounds; no effect on non-speech sound discrimination	Kostilainen et al.^36^
Maternal singing guided by music therapist	20–30 min 2x week 6 sessions	NA	<37wks GA *n* = 30	Improved alertness while awake	Palazzi et al.^34^
Maternal/paternal-led & infant-directed singing supported by music therapist	30 min 3x week 27 sessions max	NA	<35wks GA *n* = 208	No effect on maternal-infant bonding, parental anxiety or maternal depression signs	Gaden et al.^35^
Music therapist guided maternal singing during kangaroo care	40–45 min 2x week 4 weeks	NA	≤32wks GA *n* = 21	Increased infant mismatch responsess, suggesting heightened neural responses to changes in auditory stimuli	Partanen et al.^37^
**Music**					
Separate live music interventions: (1) lullaby, (2) ocean disc and (3) gato box applied by music therapist	10 min per intervention 3x week 2 weeks	55–65 dBA	≥32wks GA *n* = 272	Decreased heart rate, respiratory rate and parental stress; improved sucking behavior, caloric intake and time in active sleep	Loewy et al.^41^
Brahm’s lullaby	15 min sessions 3 consecutive days postnatal age of ≥3 days	65 dB	29–34wks GA ≥ 3 days postnatal age ≤2800 g weight *n* = 45	Delayed improvement in oxygen saturation	Jabraeili et al.^31^
Brahms’ lullaby (Chloe Agnew’s lyrical version)	6 h	58 dB	32–36 6/7wks GA *n* = 30	Higher Burdjalov Scores; decreased number of interruptions during quiet sleep (QS); no effect on number of QS cycles per hour, median QS duration or ratio of QS time during study	Stokes et al. 2017
Live pentatonic music on children’s harp	15 min	NA	26–34 6/7wks GA *n* = 19	Increased heart rate variability; no effect on number of desaturations, heart rate or respiratory rate	Ranger et al.^40^
Developmental music therapy sessions conducted by music therapist	25–30 min per session Group A: 2x week 4 weeks Group B: 4x week= weeks 1/3 0x= weeks 2/4	NA	44–66wks CGA *n* = 24	Comparable improvement in developmental milestone scores with both interventions	Emery et al. 2018
Vollenweider musical piece played through headphones	8 min 33wks CGA to discharge	NA	<32wks GA *n* = 30	Increased microstructural maturation in white matter neural tracts involved in auditory & emotional processing and increased amygdala volumes	Sa de Almeida et al.^7^
Individual improvised singing performed by music therapist	10–50 min 2x week 2nd week of life until discharge	NA	<32wks GA *n* = 40	Decreased respiratory rate and increased oxygen saturation during sleep	Kobus et al.^42^
Pain management	Varied	NA	<34wks GA	Reduced Premature Infant Pain Profile scores and stress levels; improved oxygen saturation levels	Ou et al. 2024
**Womb Sounds**					
Intermittent, rhythmic, womb-like low frequency (500–1000 Hz) sounds	6 h sessions 2x in 24 h	65–70 dB	≤32–36wks CGA *n* = 25	Decreased bradycardic events during first 6 h of intervention and hypoxemia episodes during day-time exposure; no effect on the number of apneic events, HRV or salivary cortisol levels	Parga et al. 2018
Womb sounds	5 min before and during procedure	50 dB	31–36wks GA *n* = 32	Decreased pain severity, stress and crying time, and increased comfort levels and oxygen saturation	Kahraman et al.^23^
Synthesized maternal heartbeat sounds superimposed on light rain sounds	30 min per day 14 days	34–45 dB	≥27 and <37wks GA *n* = 121	Improved behavioral state, and inceased weight gain and average daily milk intake for first 2 weeks of intervention; decreased heart rate	Zhang et al. 2022
**Multiple Auditory Interventions**					
Maternal sound recording (speaking, reading, singing and heartbeat sounds)	30 min 4x in 24 h 7th day of life until discharge	55–60.6 dBA	26–32wks GA *n* = 14	Decreased number of cardiorespiratory events	Doheny et al.^5^
Biological maternal sounds (BMS) including speaking, reading and singing and heartbeat sounds	45 min 4x in 24 h first 28 days of life	<65 dBA	≥25 & ≤33wks GA BW ≥ 700 and ≤1500 g *n* = 32	Increased weight gain and weight gain velocity; no effect on duration of NPO, full enteral feeds or total fluid and caloric intake	Zimmerman et al.^29^
Pacifier-activated music (PAM) player programmed to play maternal voice (reading) and singing	15 min 5x per day 5 consecutive days	NA	34–35 6/7wks CGA *n* = 94	Improved oral feeding rate, volume of oral intake and suck pressure	Chorna et al.^32^
Pacifier-activated music (PAM) player programmed to play maternal voice (reading) and singing	15 min 5x per day 5 consecutive days	NA	34–35 6/7wks CGA *n* = 72	No negative affect on developmental progression or feeding-related progress during 1st year of life	Hamm et al.^33^
Maternal voice (speaking, reading and singing) & heartbeat sounds	30 min 4x a day 24 sessions during 1st month of life	57.2 dBA	25–32wks GA *n* = 20	Decreased heart rate throughout 1st month of life	Rand et al.^30^
Maternal sounds (speaking, reading and singing)	45 min 4x per day 1st month of life	≤65 dBA	25–32wksGA *n* = 40	Increased bilateral auditory cortex	Webb et al.^8^
Recorded maternal singing and heartbeat duirng recovery post retinopathy of prematurity exam	5 min	60 dBA	31–36 6/7wks CGA < 2500 g *n* = 97	Increased rate at which Premature Infant Pain Profile scores return/decrease to baseline	Corrigan et al.^9^
Family-centered music therapy (Ocean Disc & breathing, humming melodic patterns and singing), guitar, during skin-to-skin contact	30–45 min 2 sessions	50 dB	<36wks GA *n* = 68	Improved autonomic nervous system stability; no effect on parent-infant attachment or parental anxiety	Yakobson et al.^38^

*GA* gestational age, *CGA* Corrected gestational age, *MDI* motor developmental index, *HRV* Heart rate variability.

^a^NA information not available.

Although there were no risks associated with earplugs (including death before discharge, length of stay, irritation to the ear or ability to pass a hearing screen), other approaches have avoided patient contact focusing on minimizing or masking environmental sounds. For example, a recent 4 week noise reduction program placing infants in incubators, diminishing door rings and replacing phone-related noises and alarms with flashlights had no effect on salivary cortisol levels.^25^ Similarly, application of “white noise”, defined as sounds of light rain, played on an MP3 player placed by the infant’s ear to reduce harmful auditory exposures while in the incubator also had no effect on weight gain, sleep wake patterns, oxygen saturation or heart rate.^26^ In contrast, in a separate study, light rain superimposed on maternal heart sounds, particularly heartbeat sounds resulted in a reduction in heart rate and promoted physical and behavioral development.^27^ Overall, these studies suggest that reducing ambient noise by earmuffs/earplugs or patient room design (open bay versus single patient) may yield some benefit with additional studies needed to identify the optimal intervention.

### Maternal/Adult voice

One of the earliest auditory stimuli to the fetus is maternal voice and thus the newborn can discriminate his/her mother’s voice immediately after birth.^4,21^ The effect of live or maternal voice recordings on stress related benefits in the NICU setting varies (Table 1). Some studies have shown improved oxygen saturation, increased comfort during painful procedures,^23^ reduced heart rate and a more stable skin color^28^ while others have shown no effect on salivary cortisol levels, heart rate and oxygen saturation.^26^

Maternal voice may also induce maturation of both cognitive and respiratory control centers of the brain. For example, early exposure to maternal and/or adult voices improved visual attention performance,^28^ and general motor scores.^28^ during term equivalent and neurofunctional assessments at 3, 6, 8 and 18 months^6^ corrected age suggesting long term benefits. Exposure to combined stimuli using maternal voice (including speaking, reading, and singing) enlarged bilateral auditory thickness^8^ suggesting overall enhanced brain growth. Furthermore, maternal voice superimposed with heartbeat sounds improved weight gain,^29^ decreased heart rate,^30^ and improved cardiorespiratory outcomes in infants >33 weeks corrected age,^5^ perhaps indicating there is an “optimal” therapeutic window where brain development is most susceptible to auditory stimulation.

### Maternal singing

Maternal singing includes both the ability of preterm infants to differentiate between their mothers’ voice and the potential added benefit of frequencies and rhythmic wave forms thought to reduce neonatal stress (Table 1). For example, in a study conducted on preterm infants of 29–34 weeks gestation and <2800 g, maternal-sung lullabies improved oxygen saturation 15 min after the music exposure.^31^ In another study, when recordings of maternal singing combined with heartbeat sounds were played following eye exams to detect retinopathy of prematurity, the rate at which Premature Infant Pain Profile scores returned down to baseline increased.^9^ suggesting that music therapy may promote recovery following painful procedures.

To reduce concerns of irritability, some studies have implemented infant-dependent interventions, allowing the infant to decide the duration of exposure by utilizing pacifier-activated music (PAM) players. When the PAM player was programmed to play mothers’ voice, suck pressure, volume of oral intake and the amplitude and rate of swallowing during oral feeding all improved^32^ with no long-term detrimental effects on oral feeds^33^ suggesting that pacifier activated music may provide positive feedback leading to improved feeding patterns in preterm infants.

The presence of a music therapist guiding auditory interventions may further impact infant outcomes. In a study of preterm infants of <37 weeks gestational age, 6 music therapy sessions conducted by a music therapist and supporting maternal singing augmented eye opening frequency, suggesting increased infants’ engagement during the time they are awake despite similar durations of maternal speech and singing between the control and intervention group.^34^ In contrast, in a separate study of parent-led, infant-directed singing guided by a licensed musical therapist (3x per week with up to 27 sessions) had no effect on mother-infant bonding.^35^ When repeated music therapist guided maternal singing was combined with kangaroo care/skin-to-skin contact (until the infant reached 40 weeks corrected age), infant mismatch responses (MMRs), a biomarker of auditory cortex detection to changes in stimuli, increased in response to speech sounds.^36^ This study was followed by a similar study of maternal singing/kangaroo care with and without a music therapist, resulting in increased MMRs in the former group suggesting heightened neural changes with the addition of a music therapist.^37^ Lastly, family-centered music therapy, involving the direction of a musical therapist in singing simple, melodic patterns with instrumental accompaniment, improved autonomic nervous system stability of the preterm infant (as measured by changes in high frequency power of heart rate variability), although parent-infant attachment was not significantly changed.^38^ Taken together, these studies suggest that music therapist instruction before and/or during auditory and multisensory interventions alike may promote infant engagement and auditory cortex activation but do not seem to improve parent-infant bonding. While it is important to recognize the diverse features among these studies, from a more holistic viewpoint, these study protocols suggest that the addition of a music therapist is feasible and potentially effective.

### Music

**Fig. 2:**
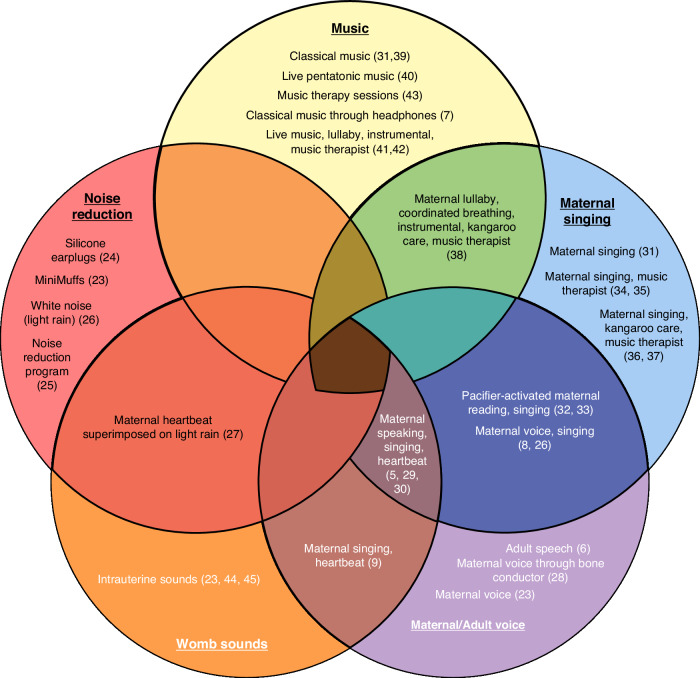
Venn diagram representing the five categories of auditory interventions. Overlaps indicate that the specific exposure involved multiple types of interventions simultaneously.

Classical and/or instrumental music is one of the most researched auditory interventions for the preterm infant (Table 1, Fig. 2), most likely because of its positive connotations for generalized development among the public. The effect of music on outcomes in preterm infants may depend on the musical piece, mode of delivery and duration of exposure. For example, 6 h recordings of a lyrical version of Brahm’s Lullaby (played at <60 dB) elicited more mature active sleep-wake patterns as measured by aEEG recordings and higher Burdjalov scores.^39^ Exposure to the Brahm’s lullaby also improved oxygen saturation levels 15 min after the intervention.^31^ In contrast, live pentatonic music on the children’s harp had no effect on oxygen desaturation frequency, heart rate or respiratory rate but increased heart rate variability parameters associated with improved autonomic nervous system maturity.^40^ Lastly, an 8-minute original piece by Andreas Vollenweider played through headphones 5 times a week from 33 weeks corrected age until discharge led to significant improvements in neurodevelopmental outcomes in preterm infants, manifesting as increased white matter maturation in the acoustic/auditory radiations, external capsule/claustrum/extreme capsule and uncinate fasciculus structures of the brain, along with larger amygdala volumes.^7^

Like maternal and family sourced interventions, the effects of music exposures may be optimized by the guidance of a certified music therapist. Loewy et al.^41^ investigated the effects of three interventions: (1) a lullaby melody chosen by a parent, (2) the gato box, a wooden instrument meant to mimic attenuated heartbeat sounds neonates hear in the womb and (3) the ocean disc, a circular instrument with metal balls meant to create a sound mimicking that of the womb sound environment.^41^ Heart rates decreased during the lullaby and gato box, with a reduction in heart rate following all interventions. The gato box increased sucking during feeds while the ocean disc increased time in active sleep and decreased respiratory rate. In a separate study, music therapists performed individual, improvised singing and/or the use of the sansula, an instrument of similar appearance to a wooden ring that plays soft sounds. These interventions resulted in a decrease in respiratory rate and an accompanying increase in oxygen saturation during sleep.^42^ These studies suggest that responses to stimuli may be dependent on the frequency and rhythm of the intervention.

A more complex, longitudinal study focused on implementing developmental music therapy sessions over a 4-week period by board-certified music therapists with two potential treatment options: (1) traditional therapy with sessions conducted 2 times (2x) per week compared to (2) the intermittent-intensive group receiving the sessions 4x, 0x, 4x and 0x for weeks 1–4, respectively.^43^ In terms of gross/fine motor, cognitive, emotional and communication skills, both approaches were equally effective,^43^ suggesting music therapists, families and staff can have flexibility when tailoring individual music therapy stimulations.

Lastly, a recent meta-analysis by Ou et al.^44^ examined the effect of music before painful procedures. Four randomized controlled trials met inclusion criteria with variations in music interventions including lullabies, nursery rhymes and classical music. The meta-analysis demonstrated that, overall, music interventions are effective in reducing Premature Infant Pain Profile scores and stress levels and improving oxygen saturation levels in preterm infants.

### Womb sounds

Many infant studies focus on music or maternal voice, but these may not be the optimal stimuli especially in the case of the preterm infant who has been deprived of *in utero* auditory conditions in the last trimester (Table 1). Mother’s heartbeat recorded within 24 h of birth combined with white noise showed improved behavioral state, increased milk consumption during the first 2 weeks of intervention and weight gain.^27^ Other studies have focused on producing a more accurate replication of the entire sound spectrum experienced in the womb. Commercial recordings of intrauterine and womb-like sounds have been beneficial in improving comfort during painful procedures^23^ and decreasing the number of bradycardia events during the first 6 h of the intervention,^45^ but these may not accurately represent the actual womb environment.^46^ Using electronic stethoscopes, Parga et al.^46^ measured intra-abdominal sounds from 50 mothers during their 2nd and 3rd trimesters of pregnancy (corresponding to a fetal gestation of 13 to 40 weeks). Spectral analysis was used to identify the various sound components of the womb environment and how they change with advancing pregnancy. Accordingly, maternal recordings were characterized by periodicity of maternal and fetal heartbeat and respiration superimposed on randomly occurring, low-frequency bowel sound bursts. Abdominal enlargement with increasing gestation increased filtering in the mid (100–500 Hz) and high (500–5000 Hz) frequency bands with no effect in the lowest band of 10–100 Hz. These findings suggest that fetal exposure of auditory stimuli in the womb encompasses dynamic patterns that change with increasing gestation.

Exposure to *in utero* sounds may play an important role in neuronal maturation. For example, a study in a small cohort of 20 infants at 32–36wks corrected age showed that short-term exposure (6 h blocks over 24 h) of womb recordings stabilized cardiorespiratory patterns, reduced episodes of intermittent hypoxemia and bradycardia with no change in frequency of apneas or heart rate variability.^45^ Given the dynamic patterns of *in utero* sounds during pregnancy, future studies delivering sound to preterm babies are needed to explore individualized womb recordings matching the corrected gestational age of the preterm infant at the time of sound exposure.

## Summary

Studies using a range of postnatal auditory stimulations (Fig. 2) have suggested that exposure to sounds or lack thereof may have a significant impact on the preterm infants’ medical trajectory throughout NICU hospitalization and beyond. Single patient rooms, earplugs and/or noise attenuators foster a low to no sound environment while white noise can enhance a consistent level of neutral sound amongst potentially harmful stimuli in the NICU. Womb, heart, and respiratory sounds can encourage development due to its similarity to the maternal third trimester womb environment while musical instruments and maternal voice have unique vibrations and waveforms that can replicate fetal exposure to maternal stimuli.

Single center studies have reported a range of effectiveness of sound exposures. Composite findings from a Cochrane review focused on sound reduction in the NICU and long-term neurodevelopmental outcomes have been inconclusive,^47^ suggesting more controlled trials are needed. A separate Cochrane meta-analysis of music and vocal interventions^48^ suggests that these interventions do not increase oxygen saturation or decrease respiratory rate during and probably not after the intervention compared to infants receiving standard care. However, significant reductions in heart rate were found both during and after the auditory therapy suggesting the potential for decreasing infant stress. Both Cochrane reviews were limited by a small number of controlled trials and low certainty of evidence with a wide range of interventions in terms of type, frequency, and duration of exposure. Pertinently, there were no reports of adverse effects from music and voice.

The corrected gestational age may be an important factor to consider for determining when to implement the auditory intervention. For example, a reduction in cardiorespiratory events during maternal sound stimulation was found to be most effective for infants ≥33 weeks corrected gestational age possibly due to increased brain plasticity during this developmental window^5^ and could explain the lack of benefits seen in the meta-analysis by Haslbeck et al.^48^.

Single auditory interventions may also be inadequate and brain maturation may require multi-sensory inputs to address growth of complex neuronal networks. This approach is being addressed by a current ongoing trial assessing cortical multisensory processing/neurodevelopment at discharge and 1–2 years of age in 200 infants randomized to either *standard care* (non-contingent recorded parent’s voice and skin to skin contact) or *multisensory stimulation*. The intervention group includes a range of stimuli including holding and light pressure containment (tactile), playing of the mother’s voice contingent on the infant’s pacifier sucking (auditory), exposure to a parent-scented cloth (olfactory) and exposure to carefully regulated therapist breathing that is mindful and responsive to the child’s condition (vestibular). These interventions are given 20 times over 2–3 weeks.^49^ The results of this trial may provide insight into optimal approaches of invoked therapies during the NICU stay.

Lastly, the specific mechanisms involved in auditory induced plasticity have yet to be elucidated. Infant mismatched responses (MMRs), EEGs, auditory evoked potentials and neuroimaging techniques have shown quantifiable alterations in brain plasticity structure and function and may provide further insight into potential mechanistic pathways related to auditory induced improvements in long term neurodevelopmental outcomes.^50^

In summary, studies focusing on auditory stimulation during early postnatal life of preterm infants suggest potential benefits in brain maturation and reduced pain scores but lack reliable and consistent findings. Future and ongoing studies examining the various modalities of auditory stimulation can provide guidance on the optimal type, sound level, and window of exposure to foster maturation in preterm infants deprived of the nutrient-rich, protective environment of the womb during later stages of development.
